# Development and validation of a FACS-based lipoprotein localization screen in the Lyme disease spirochete *Borrelia burgdorferi*

**DOI:** 10.1186/1471-2180-10-277

**Published:** 2010-11-03

**Authors:** Ozan S Kumru, Ryan J Schulze, Joyce G Slusser, Wolfram R Zückert

**Affiliations:** 1 Department of Microbiology, Molecular Genetics and Immunology, University of Kansas Medical Center, Mail Stop 3029, 3901 Rainbow Boulevard, Kansas City, KS, 66160, USA; 2 University of Bristol, Department of Biochemistry, School of Medical Sciences, University Walk, Bristol BS8 1TD, UK

## Abstract

**Background:**

In our previous studies on lipoprotein secretion in the Lyme disease spirochete *Borrelia burgdorferi*, we used monomeric red fluorescent protein 1 (mRFP1) fused to specifically mutated outer surface protein A (OspA) N-terminal lipopeptides to gather first insights into lipoprotein sorting determinants. OspA:mRFP1 fusions could be detected by epifluorescence microscopy both in the periplasm and on the bacterial surface. To build on these findings and to complement the prior targeted mutagenesis approach, we set out to develop a screen to probe a random mutagenesis expression library for mutants expressing differentially localized lipoproteins.

**Results:**

A Glu-Asp codon pair in the inner membrane-localized OspA20:mRFP1 fusion was chosen for mutagenesis since the two negative charges were previously shown to define the phenotype. A library of random mutants in the two codons was generated and expressed in *B. burgdorferi*. *In situ *surface proteolysis combined with fluorescence activated cell sorting (FACS) was then used to screen for viable spirochetes expressing alternative subsurface OspA:mRFP1 fusions. Analysis of 93 clones randomly picked from a sorted cell population identified a total of 43 distinct mutants. Protein localization assays indicated a significant enrichment in the selected subsurface phenotype. Interestingly, a majority of the subsurface mutant proteins localized to the outer membrane, indicating their impairment in "flipping" through the outer membrane to the spirochetal surface. OspA20:mRFP1 remained the protein most restricted to the inner membrane.

**Conclusions:**

Together, these results validate this FACS-based screen for lipoprotein localization and suggest a rather specific inner membrane retention mechanism involving membrane anchor-proximal negative charge patches in this model *B. burgdorferi *lipoprotein system.

## Background

Temporally and spatially regulated expression of surface-exposed lipoproteins such as OspA, OspC and VlsE enables the Lyme disease spirochete *Borrelia burgdorferi *to adapt to changing environmental conditions and allows for maintenance of the organism within an enzootic tick-mammal cycle [1-3]. Yet, we are only beginning to understand the factors that govern accurate localization of these important virulence factors to the bacterial cell surface, thereby generating the pathogen-host interface. In prior studies, we demonstrated a role for the N-terminal 'tether' region of these lipoproteins in the localization process. Fusion of the first five residues of the mature outer surface lipoprotein OspA was sufficient to target the red fluorescent reporter protein mRFP1 to the surface of the *Borrelia *cell [4]. The same study also revealed that previously identified lipoprotein sorting rules for *Enterbacteriaceae *and *Pseudomonales *[5-7] did not apply to *Borrelia *lipoproteins. An alignment of *B. burgdorferi *lipoprotein tether peptide sequences failed to reveal any apparent primary sequence conservation. Trafficking may thus depend on specific biophysical properties of the tether polypeptide such as hydrophobicity, charge, or secondary structure propensity, rather than strict amino acid identity alone [8,9]

In the present study, we designed and tested an experimental approach that might help in elucidating these still obscure sorting signals. Based on an existing OspA tether-mRFP1 fusion with a characterized inner membrane (IM) release defect, we generated a partially randomized fluorescent lipopeptide library in *B. burgdorferi*. A fluorescence-activated cell sorting (FACS)-based screen was then used to enrich for mutants localizing to the periplasm. Our results indicate that this approach can become an important tool to detect general patterns in peptides mediating surface or subsurface localization.

## Methods

### Bacterial strains and growth conditions

*Borrelia burgdorferi *B31-e2 [10] is a high passage clone of type strain B31 (ATCC 35210) and was generously provided by B. Stevenson (University of Kentucky, Lexington, KY). *B. burgdorferi *were cultured in liquid or solid BSK-II medium at 34°C under 5% CO_2 _[11,12]. *E. coli *strains TOP10 (Invitrogen, Carlsbad, CA) and XL10-Gold (Stratagene) were used for recombinant plasmid construction and propagation and grown in Luria-Bertani Lennox broth (LB) or on LB agar (Difco). Unless otherwise specified, all bacterial cultures were supplemented with kanamycin (Sigma-Aldrich) at concentrations of 30 μg ml^-1 ^or 200 μg ml^-1 ^in *E. coli *or *Borrelia*, respectively.

### Construction of mutant plasmid library

**Figure 1:**
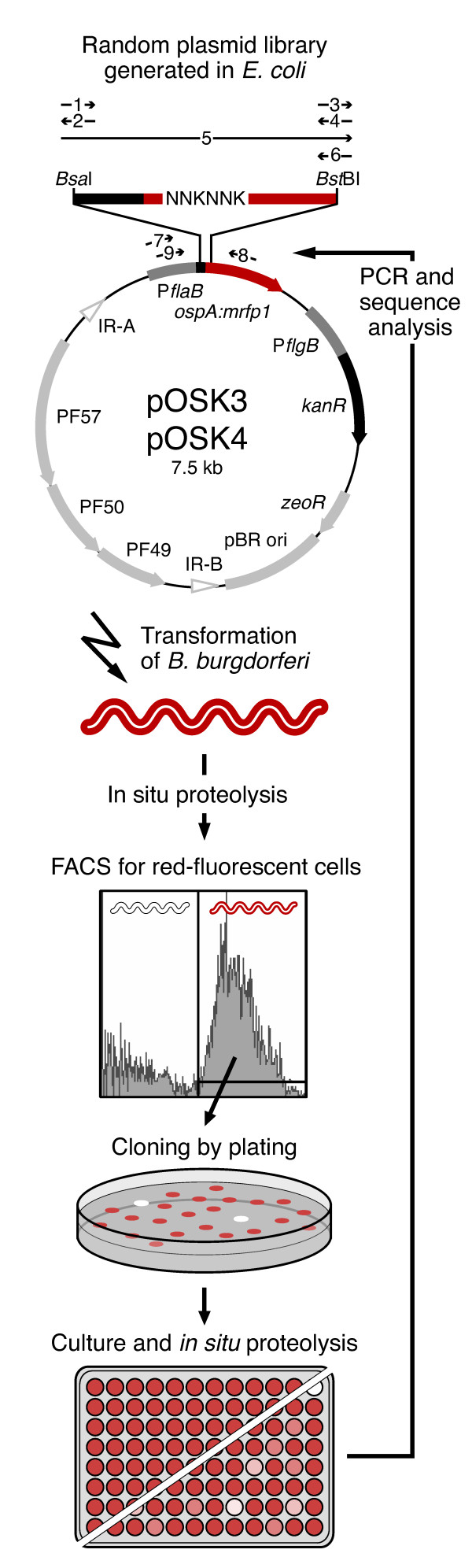
**Screening strategy for subsurface OspA:mRFP1 fusions**. A random mutagenesis oligo was synthesized to change mRFP1 codons E4 and D5 in OspA20:mRFP1 to any amino acid, with a bias against stop codons (except for amber UAG, see text). The oligo was converted to a double-stranded linker and ligated with a shuttle vector carrying the 5' and 3' portions of the OspA20:mRFP1 fusion gene. The resulting library was amplified in *E. coli *and used to transform *B. burgdorferi*. A presorted population of red fluorescent spirochetes was incubated with proteinase K, washed, and sorted again for red fluorescence. Clones grown from individual colonies were grown in 96-well plates and subjected to a confirmatory *in situ *proteolysis assay. PCR and DNA sequence analysis revealed the mutant genotypes. Numbered arrows indicate specific oligonucleotides used (Table 1). For details, see the Materials and Methods section.

First, translationally silent restriction endonuclease sites for *Bsa*I and *Bst*BI were engineered into plasmids pRJS1016 and pRJS1009 [4] using the QuickChange II XL site-directed mutagenesis kit (Stratagene) and oligonucleotide primers BsaImut-fwd and -rev and Bstmut-fwd and -rev (IDT Integrated DNA Technologies, Coralville, IA) to yield pOSK1 and pOSK2, respectively (Figure 1 and Table 1). Next, a 114-mer random mutagenesis oligonucleotide, Rmut-oligo, was synthesized and purified by polyacrylamide gel electrophoresis (PAGE, Integrated DNA Technologies, Coralville, IA). In Rmut-oligo, the mRFP1 E4 and D5 codons are replaced by NNK. K, i.e. G or T in the third position allows for any amino acid, but is biased against stop codons. Only the UAG "amber" codon had to be allowed to cover all amino acids. Rmut-oligo was converted into a double-stranded DNA molecule using oligonucleotide Rmut-rev and the large fragment of DNA polymerase I (Invitrogen). The fill-in reaction was terminated using a MinElute reaction cleanup kit (Qiagen). pOSK1 or -2 and the double-stranded Rmut linker were then both digested with *Bsa*I and *Bst*BI (New England Biolabs). The cut vectors were treated with shrimp alkaline phosphatase (Invitrogen) before ligation to the Rmut DNA linker with a Quick Ligation kit (NEB), yielding pOSK3 and -4, respectively. Chemically competent *E. coli *Top10 were transformed with approximately 10 ng of the ligation reaction and transformants were grown in batch in 500 ml of LB broth for 18 hours at 37°C with aeration. Plasmid DNA was then isolated using a Biotech Spin Doctor BAC prep kit (Midwest Scientific) following the manufacturer's protocol. *Borrelia *cells were transformed by electroporation with 2 μg of plasmid DNA using established protocols [13,14] and grown in liquid BSK-II media at 34°C and 5% CO_2_.

**Table 1:** Oligonucleotides used in this study

Number^a^	Name	Target/Purpose	Sequence (5' to 3')^b^
1	Bsamut-fwd	Introduction of silent mutation in OspA L10 codon yielding *Bsa*I site	GGGAATAGGTCT**C**ATATTAGCCTTAATAGC
2	Bsamut-rev	Introduction of silent mutation in OspA L10 codon yielding *Bsa*I site	TGCTATTAAGGCTAATAT**G**AGACCTATTCC
3	Bstmut-fwd	Introduction of silent mutation in mRFP1 V15R16 codons yielding *Bst*BI site	TGCGCTTCAAGGT**T**CG**A**ATGGAGGGCTCCG
4	Bstmut-rev	Introduction of silent mutation in mRFP1 V15R16 codons yielding *Bst*BI site	GGAGCCCTCCAT**T**CG**A**ACCTTGAAGCGCATGAAC
5	Rmut-oligo	Random mutagenesis oligo	TATTTATTGGGAATAGGTCTCATATTAGCCTTAATAGCATGTAAGCAAAATGCCTCCTCC**NNKNNK**GTCATCAAGGAGTTCATGCGCTTCAAGGTTCGAATGGAGGGCTCCGTG
6	Rmut-rev	Generation of double-stranded DNA from Rmut-oligo	CACGGAGCCCTCCATTCGAACC
7	Mutscreen-fwd	Amplification of mutated ospA:mrfp1 region from P*flaB*	ATGCTATTGCTATTTGCGTTTC
8	Mutscreen-rev	Amplification of mutated ospA:mrfp1 region from *ospA*	ATGGTCTTCTTCTGCATTAC
9	Mutscreen-seq	Sequencing of amplified ospA:mrfp1 region from P*flaB*	AAAGGATTTGCCAAAGTCAG

^a^Numbers correspond to primer numbers indicated in Figure 1.

^b^Introduced restriction sites are underlined; mutated nucleotides are in bold.

### Fluorescence activated cell sorting (FACS)

2 × 10^6 ^spirochetes were harvested as described [4], washed twice with phosphate buffered saline containing 5 mM MgCl_2 _(PBS+Mg), and incubated with a final concentration of 50 μg ml^-1 ^proteinase K (Invitrogen) for one hour at room temperature. Mock-treated cells were incubated in PBS+Mg only. Cells were then washed three times with PBS containing 0.1% bovine serum albumin (PBS+BSA) and resuspended in 1 ml of PBS+BSA at a density of 1 to 1.5 × 10^6 ^cells ml^-1^. Spirochetes retaining red fluorescence were then sorted by FACS on a BD FACSAria (BD Biosciences) at a flow rate of 200 events s^-1 ^and 55 psi through a 70 μm nozzle. Excitation, long pass, and band pass wavelengths were 488 nm, 635 nm, and 695 ^+/- ^40 nm, respectively. Upon completion of FACS, the volume of the sorted cells (about 1 ml) was immediately adjusted to 12 ml with BSK-II and incubated at 34°C. The FlowJo program suite, version 7.2.2 (Treestar), was used for data analysis.

### DNA sequence analysis and identity of subsurface retention signals

Spirochetes were counted using a Petroff-Hauser counting chamber, adjusted to 200 cells ml^-1^, plated on solid BSK II media [12], and incubated at 34°C and 5% CO_2_. Individual colonies were picked using sterile toothpicks and cultured in 200 μl of BSK-II complete media in a sterile 96-well tissue culture plate (Corning). The mutated *ospA-mrfp1 *region was amplified from 1 μl of 1:10 diluted culture in sterile water using primers Mutscreen-fwd and -rev (Figure 1 and Table 1). PCR products were purified using a PCR purification kit (Qiagen) and sequenced (AGCT Inc., Wheeling, IL) using primer Mutscreen-seq. Each sequenced mutant was cultured in liquid BSK-II culture for further analysis.

### Protein localization assays

To assess protein surface exposure by protease accessibility intact *B. burgdorferi *cells were treated *in situ *with proteinase K as described [4,15]. In order to determine localization of mRFP1 outer membrane vesicles were isolated and purified by treatment of *B. burgdorferi *cells with low pH, hypotonic citrate buffer followed by isopycnic sucrose gradient ultracentrifugation as described [4,16].

### Protein gel electrophoresis and immunoblot analysis

Proteins were separated by sodium dodecyl sulfate-12.5% or -10% polyacrylamide gel electrophoresis (SDS-PAGE) and visualized by Coomassie blue staining. For immunoblots, proteins were electrophoretically transferred to a Immobilon-NC nitrocellulose membrane (Millipore) using a Transblot semi-dry transfer cell (Bio-Rad) as described. Membranes were rinsed in 20 mM Tris-500 mM NaCl, pH 7.5 (TBS). TBS with 0.05% Tween 20 (TBST) containing 5% dry milk was used for membrane blocking and subsequent incubation with primary and secondary antibodies; TBST alone was used for the intervening washes. Antibodies used were anti-mRFP1 rabbit polyclonal antiserum ([17]; 1:5000 dilution, a gift from P. Viollier, Case Western Reserve University, Cleveland, OH), anti-OppAIV rabbit polyclonal antiserum ([18]; 1:100 dilution, a gift from P.A. Rosa, NIH/NIAID Rocky Mountain Laboratories, Hamilton, MT) and anti-FlaB rabbit polyclonal antiserum ([19]; 1:1000 dilution; a gift from M. Caimano, Univ. of Connecticut Health Center, Farmington, CT), or anti-OspA mouse monoclonal ([20]; H5332; 1:50 dilution). Secondary antibodies were alkaline phosphatase-conjugated goat anti-rabbit IgG (H+L) or goat anti-mouse IgG (H+L) (Sigma). CDP-Star (GE Healthcare Life Sciences) was used as the alkaline phosphatase substrate for chemiluminescent detection. Restore Western blot stripping reagent (Pierce) was used to remove bound antibodies from immunoblots to allow for reprobing of membranes.

### Densitometry and calculations

Densitometry of Coomassie blue-stained protein bands and Western blot signals acquired with a Fuji LAS-4000 fluorescence imager with a linearity of 4 orders of magnitude was done using the Image J image analysis software http://rsb.info.nih.gov/ij/. The percentage of surface-localized protein was calculated using the following formula: % surface = 100 - [(mRFP1_+pK _x FlaB_-pK_) ÷ (mRFP1_-pK _x FlaB_+pK_)] × 100, where mRFP1 and FlaB indicate the raw Western immunoblot densitometry data in absence (-pK) or presence (+pK) of proteolysis. Negative % surface values obtained for four mutants (ED, SK, TR and GR) were set to zero. The OM/PC distribution ratio using the following formula: ratio_OM/PC _= (mRFP1_OM _÷ mRFP1_PC_) ÷ [(OspA_OM _÷ OspA_PC_) - (OppAIV_OM _÷ OppAIV_PC_)], where mRFP1, OspA and OppAIV represent the raw Western immunoblot densitometry data in either the OM or PC fractions. Genomic *B. burgdorferi *strain B31 (GenBank Accession # NC_001318) codon usage data were acquired from the Georgia Tech Codon Usage Database http://exon.gatech.edu/GeneMark/metagenome/CodonUsageDatabase/ and compared to detected protein levels. Codon usage-to-protein level correlation coefficients were calculated using Microsoft Excel for Mac 2008.

## Results & Discussion

### Design of a fluorescence-based screen for lipoprotein localization in *B. burgdorferi*

In our recent studies, the use of fusions of red fluorescent mRFP1 to various N-terminal fragments and point mutants of *B. burgdorferi *surface lipoprotein OspA led to an initial assessment of the sequence requirements for proper surface display [4,21]. To complement this step-wise, targeted mutagenesis approach, we set out to develop a random mutagenesis screen. Our starting point was a previously described OspA-mRFP1 fusion, OspA20:mRFP1, which could be redirected from the IM to the bacterial surface by mutagenesis of two adjacent negatively charged amino acids (Glu-Asp) at the N-terminus of mRFP1 to two Ala residues. We therefore hypothesized that (i) additional mutagenesis in this OspA20:mRFP1 dipeptide would reveal the specificity of periplasmic, particularly IM retention signals in this model lipoprotein, and that (ii) periplasmically localized fusion protein mutants could be enriched by a combination of *in situ *surface proteolysis and fluorescence-activated cell sorting (FACS). The approach is detailed in the Materials & Methods section and shown in Figure 1.

**Figure 2:**
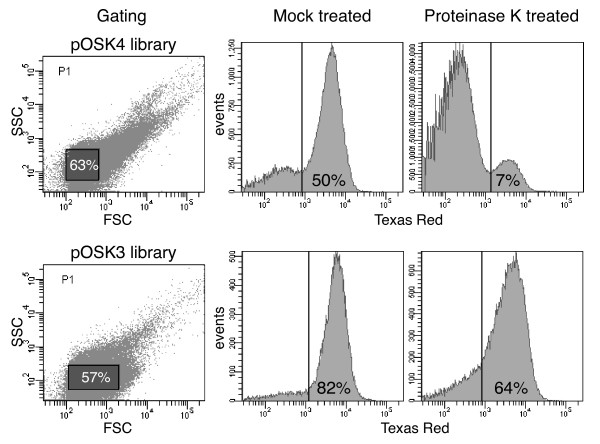
**FACS plots of OspA:mRFP1 mutant populations**. Both pOSK4 (pRJS1009-based) and pOSK3 (pRJS1016-based) *B. burgdorferi *libraries were assayed. The two panels to the left indicate the gating used. Forward scatter (FSC) is plotted against side scatter (SSC). The percentage of events, i.e. cells inside the gated population (shaded rectangles) is indicated. The four panels to the right show the distribution of presorted, i.e. OspA:mRFP1-expressing fluorescent cells upon treatment with proteinase K. Mock treated cells were incubated in buffer only. Fluorescence measured via a Texas Red filter is plotted against number of events, i.e. cells. The vertical line indicates the cut-off fluorescence for sorting. The percentage of events within the fluorescent population is indicated.

Two plasmid libraries were generated from two different starting materials, pRJS1009 and pRJS1016 [4]. pRJS1009 carried a fusion of the full-length signal peptide and tether of OspA to mRFP1 (OspA28:mRFP1), which was targeted to the bacterial surface. In pRJS1016, the OspA tether sequence was truncated to 4 amino acids (OspA20:mRFP1), which led to significant retention of the fusion in the inner spirochetal membrane. In both plasmids, a fragment containing the 5' *ospA:mrfp1 *sequence was swapped for a DNA fragment randomized at the Glu-Asp codons. After library expansion in *E. coli *and electroporation of *B. burgdorferi*, transformants were grown in liquid medium selecting for the library plasmids. To eliminate any non-expressers, we subjected the populations to a first round of FACS, collecting only cells with a clear red fluorescent signal (not shown). Gating was determined by plotting logs of forward scatter (FSC) versus side scatter (SSC) as described [22] (Figure 2). After presorting, cells were allowed to recover in liquid medium and then subjected to proteolytic shaving using proteinase K. We surmised that treated cells would remain fluorescent only if they expressed a subsurface mutant of the OspA:mRFP1 fusion.

### Genotypic and phenotypic analysis of pre- and post-sorting cell populations

**Figure 3:**
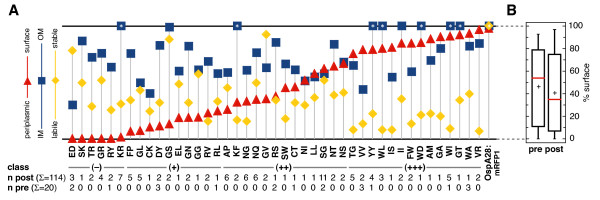
**Composite phenotypes of lipoprotein mutants**. (A) Expression, surface exposure and membrane fraction ratio values are plotted for each of the 43 identified mutants, including OspA20:mRFP1 (ED), as well as the OspA28:mRFP1 control are plotted. Data were derived from independent duplicate or triplicate Western immunoblot experiments. Representative data are shown in Figures 4, 5 and 6. Numerical data are listed in Additional File 1-Table S1. Y-axis ranges were 0-100% for expression/stability levels (yellow diamonds) and surface exposure (red triangles), and 0 to 1.0 for the OM/PC ratio (blue squares). Data points with asterisks (*) lay beyond the y-axis ranges (Additional File 1-Table S1). Mutant-specific amino acid sequences are listed in single letter code on the × axis. n indicates the number of times a particular mutant was isolated from the unsorted (pre) and sorted (post) population. Unanalyzed mutants are listed in Additional File 1-Table S1. (B) Boxplots of surface percentage values of the unsorted (pre) and sorted (post) populations. For each dataset, the box outlines the first and third quartiles, the horizontal red line indicates the median, and the vertical lines extend to the minimum and maximum values.

Compared to mock-treated cells, the fluorescent population post-treatment decreased for both libraries, suggesting that proteolytic shaving indeed resulted in a reduction of surface-associated fluorescence. Interestingly, the reduction was more significant in the pRJS1009-based library (from 50% to 7%) than the pRJS1016-based library (from 82% to 64%) (Figure 2). We initially attributed this to the potential of bleed-through of the original plasmid in the pRJS1016-derived library. Yet, further analysis showed that this effect was negligible as only three Glu-Asp clones were recovered post-sorting (see below and Figure 3).

A total of 172 random clones from the pRJS1016-derived library were analyzed by DNA sequencing. 38 clones were from a population sampled prior to proteolytic shaving and sorting (unsorted), and 134 clones were from a population sampled after proteolytic shaving and sorting (sorted). 63 mutants were identified, 8 being unique to the unsorted population, 40 unique to the sorted population, and 15 common to both populations. Within the sorted population, the majority of the mutants (40 out of 55, i.e. 73%) were recovered repeatedly, e.g. 11 times for Ser-Gly (Figure 3A and Additional File 1-Table S1). This suggested that we were approaching saturation in this experimental setting. As predicted, sorting for fluorescent cells significantly selected against the presence of non-expressing cells: the incidence of "amber" stops within the two mutated codons was reduced 18-fold, from 5 clones in the unsorted to 1 in the sorted population.

We randomly chose 93 clones from the sorted population for further analysis. This cohort covered 43 individual mutants, 11 of which were also identified in the presorted population (Figure 3A as well as Additional File 1-Table S1). The mutants were assessed for (i) protein levels and (ii) protein localization within the spirochetal cell envelope by *in situ *proteolysis and membrane fractionation. The observed protein levels provided a measure of fusion protein stability *in vivo*, as expression of all mutant proteins was driven by an identical promoter. Furthermore, there was no correlation between the genomic frequency of the introduced codons and protein levels; correlation coefficients were -0.06 and -0.30 for the first and second codon, respectively.

**Figure 4:**
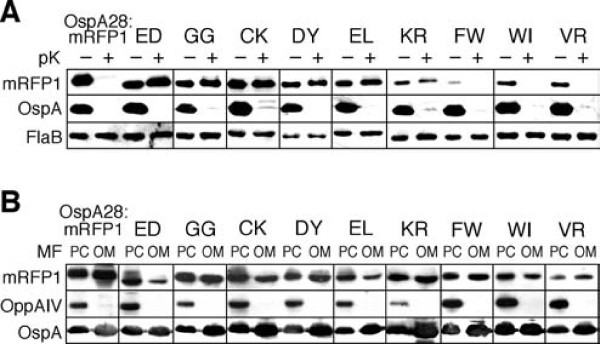
**Phenotypical analysis of select OspA:mRFP1 fusion mutants**. Representative Western blots of select mutants are shown (see Additional File 2-Figures S1 and S2 for full data set). Mutant-specific amino acid sequences are listed in single letter code above the blots. OspA28:mRFP1 and OspA20:mRFP1 (ED) were included as controls. (A) Protein expression and protease accessibility. Whole cell lysates of *B. burgdorferi *expressing mutant OspA:mRFP1 fusions from an identical P*_flaB _*promoter (Figure 1) were obtained before (-) or after (+) *in situ *treatment with proteinase K (pK). A polyclonal antiserum against mRFP1 was used to detect the OspA:mRFP1 fusions. Constitutively expressed periplasmic FlaB was used as a control for loading (to normalize signals within samples) as well as for subsurface localization (negative control). OspA served as a surface control. Untreated (-pK) samples were used to assess protein expression/*in vivo *stability of OspA:mRFP1 fusions. (B) Distribution of proteins to inner or outer membranes. Protoplasmic cylinder (PC) and outer membrane vesicle (OM) fractions from *B. burgdorferi *expressing mutant OspA:mRFP1 fusions were probed with a polyclonal antiserum against mRFP1 to detect the OspA:mRFP1 fusions. IM-localized lipoprotein OppAIV was used as a PC-specific control. Surface lipoprotein OspA was used as an outer membrane control. Note that the PC fraction also contains intact cells, i.e. also contains OM proteins.

All experiments were done in triplicate. Mutant phenotypes are summarized in Figure 3A and Additional File 1-Table S1. Figure 4 shows a representative raw dataset of mutants discussed in more detail below, while raw data for all 43 mutants can be found in the Additional Files (Additional File 2-Figures S1 and S2). OspA28:mRFP1 and OspA20:mRFP1 (labeled as ED in all figures and tables) were included as controls. Surface localization of the OspA:mRFP1 mutants was assessed by proteolytic shaving with proteinase K followed by Western immunoblotting of whole cell lysates (Figure 4A and Additional File 2-Figure S1). OspA served as a surface control while FlaB served as both a loading and periplasmic control. The signals from the OspA:mRFP1 fusion proteins were quantified by densitometry of digital fluorometric images and normalized to both OspA and FlaB signals. Analysis of the untreated whole cell lysates (lanes labeled pK- in Figure 4A and Additional File 2-Figure S1) was also used to assess OspA:mRFP1 fusion lipoprotein stability. The OspA:mRFP1 fusion protein signals were normalized to the FlaB signals, and expression/*in vivo *stability levels were calculated in percent compared to OspA28:mRFP1. In additional blots, an OspA20:mRFP1 sample was included on each blot to normalize between individual replicates (not shown). Localization of proteins to the IM or OM was assessed by Western immunoblots of PC and OM membrane fractions, using OspA and OppAIV as membrane-specific controls and normalization standards (Figure 4C and Additional File 2-Figure S2). Note that the PC fraction contains both protoplasmic cylinders and whole cells [4,16], which explains the significant presence of OM proteins such as OspA in the PC fraction. The specific formulas used to calculate both the percentage of surface-localized protein and the OM/PC distribution ratios are described in the Materials & Methods section.

### Classification of phenotypes

Based on the *in situ *proteolysis assay data, the characterized 43 mutant lipoproteins were classified according to their surface exposure phenotype (Figure 3A and Additional File 1-Table S1): 14 mutants or 31 clones were grouped as predominantly surface-exposed (class +++), 13 mutants or 42 clones had an intermediate phenotype (class ++), and 10 mutants or 22 clones localized largely to a subsurface compartment (+). 6 mutants represented by 19 clones were indistinguishable in their proteinase K accessibility phenotype from the original OspA20:mRFP1_ED _fusion (class -). Although we observed a continuum of phenotypes from IM-retained to surface-localized lipoprotein mutants, there was an appreciable enrichment of subsurface phenotypes in the sorted population. The median surface percentage dropped from 54% in the unsorted population to 35% in the sorted population (Figure 3B). The median expression levels and OM/PC ratios were 34% and 0.7 for both the unsorted and sorted populations. This indicated that the screen did not exert a pleiotropic, but rather a specific and intended selective pressure on the surface phenotype.

Surface exposure of lipoproteins in diderm bacteria can be affected by defects in either the release from the bacterial IM or a defect in translocation through the OM. To our surprise, most mutants, including the newly identified class - and + mutants localized in significant ratios to the OM (Figure 3A and Additional File 1-Table S1). One standout mutant in that respect is the Lys-Arg mutant OspA20:mRFP1_KR_: The fusion protein fractionated to the OM comparable to the surface-exposed OspA28:mRFP1, but 99% of the total protein was protected from proteinase K (Figures 3A and 4). This indicated that this and most other mutant proteins were significantly impaired in "flipping" through the OM. Two aspects of this finding are particularly intriguing. First, we recently observed a similar predominance of OM translocation defects when disrupting a Val-Ser-Ser-Leu tetrapeptide within the tether of otherwise wild type OspA. These defects were overcome when the mutant OspA tethers were fused to mRFP1, which contains a similar N-terminal Ala-Ser-Ser-Glu tetrapeptide [4,21]. The mutations introduced in this study tangentially affect this mRFP1-derived tetrapeptide by altering the Glu residue, with similar results. For example, the introduction of Gly residues as in the OspA20:mRFP1_GG _mutant led to a defect (Figures 3A and 4) while the previously described replacement by two Ala residues did not [4]. This supports our earlier speculation that the mRFP1 tetrapeptide could functionally offset an OspA tether defect [21]. Second, the original OspA20:mRFP1_ED _retains the most profound IM-release defect phenotype. The Cys-Lys mutant OspA20:mRFP1_CK_, although comparable in membrane localization, is significantly less stable *in vivo *than OspA20:mRFP1_ED _(Figures 3A and 4). Confirming our earlier site-directed mutagenesis data [4], single negative charges as in the Asp-Tyr (OspA20:mRFP1_DY_) or Glu-Leu (OspA20:mRFP1_EL_) mutants were insufficient to quantitatively restrict a lipoprotein to the borrelial IM (Figures 3A and 4). Therefore, small patches of negative charges continue as the only identified IM retention signal for lipoproteins expressed in *Borrelia *cells, albeit in an artificial model lipoprotein setting. Further studies will be needed to identify IM retention signals of natural *B. burgdorferi *lipoproteins such as OppAIV [4,18].

With few exceptions, mutants were detected at significantly lower levels than both OspA28:mRFP1 and OspA20:mRFP1, despite being expressed from an identical promoter. Interestingly, this phenotype tended to cluster with class +++ surface-localized proteins, e.g. OspA20:mRFP1_VR_, OspA20:mRFP1_WI _or OspA20:mRFP1_FW _(Figures 3A and 4). Based on structural data on the mRFP1 parent molecule DsRed, the mutated residues coincide with the transition from the fusion protein's flexible tether to the structurally confined red fluorescent protein β-barrel [23]. Amino acid substitutions, particularly with large bulky amino acids such as Trp or Phe therefore may compromise the protein fold. Based on our recent discovery that translocation of OspA through the borrelial OM requires an unfolded conformation [21], we propose that the structural instability of mutants contributes to their ultimate surface localization.

## Conclusions

Since their inception, fluorescence-based analytical and preparative methods such as flow cytometry (FCT) and FACS have reached beyond the realm of immunology. FCT already has seen several applications in spirochetal systems, predominantly in deciphering gene regulation mechanisms [22,24,25], but also in probing membrane characteristics [26]. Various FACS-based methods such as differential fluorescence induction (DFI; [27]) have been used in different bacterial systems to identify virulence factors important for different pathogenic processes such as invasion and intracellular survival (reviewed in [28]). Building on the earlier development of recombinant DNA technology [14] and fluorescent reporter genes [4,29,30], this study expands the application of FACS to the study of protein transport mechanisms. Similar FACS-based approaches are perceivable to study secretion of other microbial proteins localizing to the host-pathogen interface. The demonstrated ability to sort live *B. burgdorferi *cells for a particular fluorescent phenotype also opens the door to DFI studies, i.e. the trapping of promoters that are active during different stages in the complex multi-host life cycle of this medically important spirochete.

## Authors' contributions

OSK carried out the majority of the experimental work, analyzed the data and participated in drafting the manuscript. RJS designed the experimental approach and participated in the experimental work, analysis and drafting of the manuscript. JGS consulted on and carried out the fluorescence activated cell sorts. WRZ conceived and supervised the study, participated in the data analysis, and wrote the manuscript. All authors read and approved the final manuscript.

## Supplementary Material

Additional file 1 **Table S1**. Phenotypes of OspA20:mRFP1 fusion mutants Click here for file

Additional file 2 Figures S1 and S2. Protease accessibility and membrane localization of OspA:mRFP1 fusion mutants. Click here for file
