# Supplementary material for: Development and validation of a FACS-based lipoprotein localization screen in the Lyme disease spirochete Borrelia burgdorferi
Source: BMC Microbiol. 2010 Nov 3;10:277. doi: 10.1186/1471-2180-10-277 (PMC2987989; doi:10.1186/1471-2180-10-277)
Supplement: Additional file 1 — Table S1. Phenotypes of OspA20:mRFP1 fusion mutants [file 1471-2180-10-277-S1.PDF]

Table S1. Phenotypes of OspA20:mRFP1 fusion mutants

Peptide Sequence[a]	Codons		n Isolated	Surface Exposure Class	% Surface Mean	% Surface SD	% Expression Mean	% Expression SD	OM/PC Ratio Mean	OM/PC Ratio SD
**ED**	GAG	GAC	3	–	**0**	18	**78**	0	**0.30**	0.06
**SK**	AGT	AAG	1	–	**0**	7	**50**	17	**0.87**	0.10
**TR**	ACG	AGG	2	–	**0**	10	**29**	6	**0.72**	0.06
**GR**	GGG	AGG	4	–	**0**	3	**52**	4	**0.76**	0.12
	GGG	CGG								
**RY**	AGG	TAT	2	–	**0**	14	**26**	10	**0.64**	0.08
**KR**	AAG	AGG	7	–	**1**	5	**31**	0	**1.37**	0.30
**FP**	TTT	CCG	5	+	**6**	18	**34**	6	**0.76**	0.34
**GL**	GGT	TTG	5	+	**7**	2	**43**	5	**0.50**	0.08
	GGG	TTG								
**CK**	TGT	AAG	1	+	**10**	38	**24**	7	**0.40**	0.05
**DY**	GAT	TAT	2	+	**11**	14	**33**	13	**0.83**	0.05
**GS**	GGT	TCG	3	+	**13**	33	**88**	21	**0.99**	0.36
**EL**	GAG	TTG	1	+	**19**	33	**49**	0	**0.60**	0.16
**GN**	GGT	AAT	1	+	**19**	12	**32**	10	**0.82**	0.20
**GG**	GGT	GGG	1	+	**19**	5	**57**	36	**0.61**	0.12
**RV**	CGT	GTG	2	+	**23**	2	**16**	8	**0.69**	0.14
**RL**	CGG	TTG	1	+	**25**	4	**34**	10	**0.58**	0.17
**AP**	GCT	CCG	6	++	**26**	33	**45**	1	**0.59**	0.13
**KF**	AAG	TTT	2	++	**32**	17	**17**	7	**1.14**	0.18
**NG**	AAT	GGT	6	++	**33**	5	**15**	8	**0.61**	0.12
**NQ**	AAT	CAG	6	++	**35**	16	**50**	11	**0.76**	0.02
**GV**	GGT	GTC	2	++	**35**	16	**92**	15	**0.63**	0.07
	GGG	GTG								
**RS**	AGG	AGT	1	++	**38**	19	**35**	7	**0.85**	0.09
**SW**	AGT	TGG	1	++	**45**	4	**18**	0	**0.66**	0.17
**CT**	TGT	ACG	1	++	**46**	4	**32**	5	**0.61**	0.11
**NI**	AAT	ATT	1	++	**52**	40	**30**	12	**0.51**	0.13
**LL**	CTT	CTG	1	++	**58**	17	**36**	7	**0.55**	0.10
**SG**	AGT	GGT	11	++	**62**	18	**52**	2	**0.58**	0.09
	TCT	GGG								
	TCC	GGT								
**NT**	AAT	ACG	2	++	**64**	17	**39**	3	**0.84**	0.14
**NS**	AAT	TCG	2	++	**66**	24	**41**	6	**0.68**	0.17
**TG**	ACG	GGG	5	+++	**75**	19	**8**	5	**0.65**	0.05
**VV**	GTG	GTT	2	+++	**79**	4	**13**	2	**0.44**	0.11
**YY**	TAT	TAT	4	+++	**79**	15	**22**	7	**1.18**	0.12
**WL**	TGG	TTG	3	+++	**80**	6	**7**	2	**1.19**	0.07
**IS**	ATT	TCT	1	+++	**81**	4	**8**	1	**0.55**	0.08
**II**	ATT	ATT	2	+++	**84**	3	**35**	15	**1.05**	0.23
**FW**	TTT	TGG	2	+++	**85**	13	**13**	0	**0.59**	0.02
**WD**	TGG	GAT	1	+++	**85**	9	**21**	7	**1.08**	0.35
**AM**	GCG	ATG	1	+++	**88**	5	**22**	3	**0.69**	0.13
**GA**	GGT	GCG	1	+++	**90**	7	**20**	2	**0.80**	0.22
**WI**	TGG	ATT	5	+++	**91**	1	**9**	2	**1.23**	0.51
**GT**	GGG	ACG	1	+++	**92**	4	**34**	12	**1.11**	0.27
**WA**	TGG	GCG	1	+++	**93**	9	**39**	13	**0.82**	0.07
**VR**	GTG	CGG	2	+++	**97**	4	**7**	2	**0.84**	0.16
**N28**	N/A	N/A	N/A	+++	**98**	1	**100**	25	**2.03**	0.40

[a]mutants not analyzed in this study (n post/n pre): EI (1/0), ET (1/0), GL (1/0), LD (1/0), NR (1/0), RM (1/0), RR (1/0), SL (1/0), VT (6/1), GQ (2/1), LR (2/2), IL (1/1),ES (0/1),NW (0/1), RI (0/1), SE (0/1), TW (0/1),VA (0/1),YF (0/1), YT (0/1).
