# Supplementary material for: Development and validation of a FACS-based lipoprotein localization screen in the Lyme disease spirochete Borrelia burgdorferi
Source: BMC Microbiol. 2010 Nov 3;10:277. doi: 10.1186/1471-2180-10-277 (PMC2987989; doi:10.1186/1471-2180-10-277)
Supplement: Additional file 2 — Figures S1 and S2. Protease accessibility and membrane localization of OspA:mRFP1 fusion mutants. [file 1471-2180-10-277-S2.PDF]

# Additional File 2

Figures S1, S2

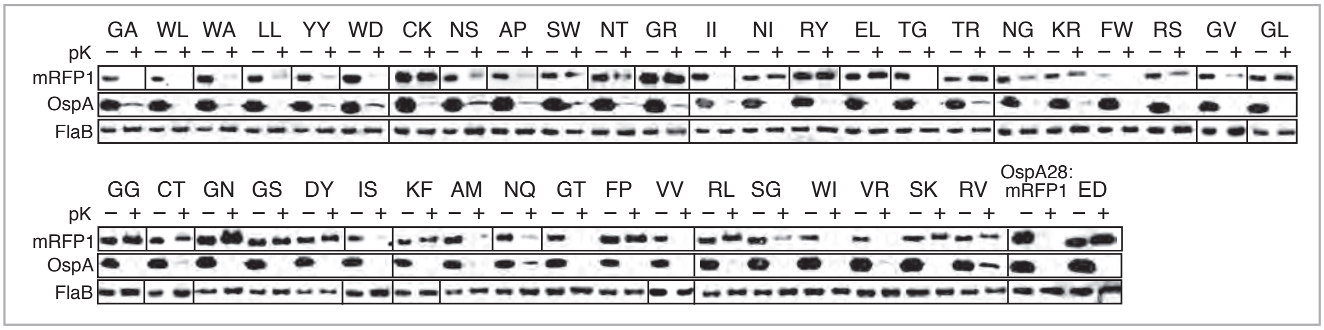


**Figure S1. Protease accessibility of OspA:mRFP1 fusion mutants.** Representative Western immunoblots of *B. burgdorferi* whole cell lysates expressing mutant OspA:mRFP1 fusions from an identical P*flaB* promoter (Fig. 1) before (–) or after (+) in situ treatment with proteinase K (pK). A polyclonal antiserum against mRFP1 was used to detect the OspA:mRFP1 fusions. Constitutively expressed periplasmic FlaB was used as a control for loading (to normalize signals within samples) as well as for subsurface localization (negative control). OspA served as a surface control. Mutant-specific amino acid sequences are listed in single letter code above the blots. OspA28:mRFP1 and OspA20:mRFP1 (ED) were included as controls.

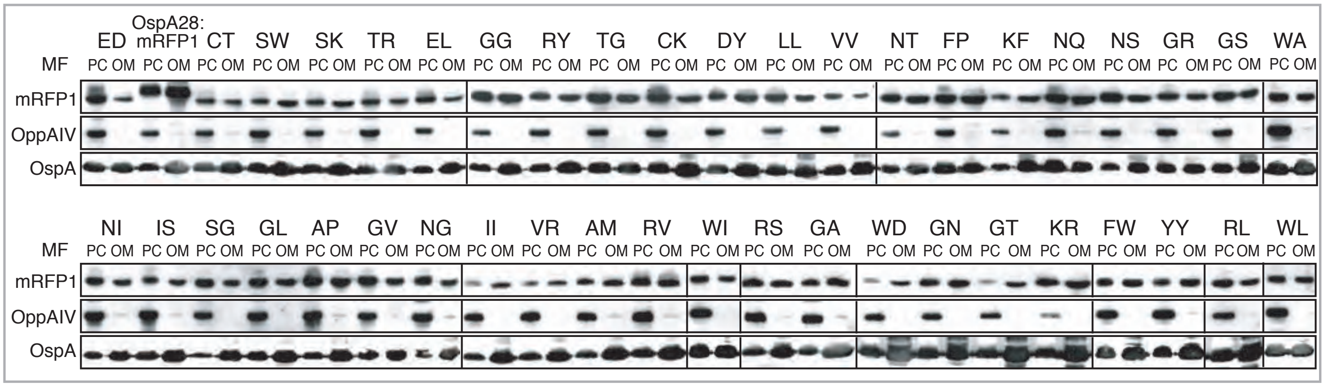


**Figure S2. Distribution of OspA:mRFP1 fusion mutants to inner and outer membranes.** Representative Western immunoblots of protoplasmic cylinder (PC) and outer membrane vesicle (OM) fractions from *B. burgdorferi* expressing mutant OspA:mRFP1 fusions. A polyclonal antiserum against mRFP1 was used to detect the OspA:mRFP1 fusions. IM-localized lipoprotein OppAIV was used as a PC-specific control. Surface lipoprotein OspA was used as an outer membrane control. Note that the PC fraction also contains intact cells, i.e. also contains OM proteins. Mutant-specific amino acid sequences are listed in single letter code above the blots. OspA28:mRFP1 and OspA20:mRFP1 (ED) were included as controls.
